# Sampling Procedures for Estimating the Infant Intake of Human Milk Leptin, Adiponectin, Insulin, Glucose, and Total Lipid

**DOI:** 10.3390/nu16030331

**Published:** 2024-01-23

**Authors:** Majed A. Suwaydi, Ching Tat Lai, Zoya Gridneva, Sharon L. Perrella, Mary E. Wlodek, Donna T. Geddes

**Affiliations:** 1 School of Molecular Sciences, The University of Western Australia, Crawley, WA 6009, Australia or msuwaydi@jazanu.edu.sa (M.A.S.); ching-tat.lai@uwa.edu.au (C.T.L.); zoya.gridneva@uwa.edu.au (Z.G.); sharon.perrella@uwa.edu.au (S.L.P.); m.wlodek@unimelb.edu.au (M.E.W.); 2 School of Applied Medical Sciences, Jazan University, Jazan 45142, Saudi Arabia; 3 ABREAST Network, Perth, WA 6000, Australia; 4 UWA Centre for Human Lactation Research and Translation, Crawley, WA 6009, Australia; 5 Department of Obstetrics and Gynaecology, The University of Melbourne, Melbourne, VIC 3010, Australia

**Keywords:** human milk, breastfeeding, lactation, hormones, adipokine, leptin, adiponectin, insulin, lipids, infant intake

## Abstract

Limited attention is given to the efficacy of protocols for the estimation of infant intake of milk components when investigating their impact on infant outcomes. We compared the actual measured intake of human milk components with estimations derived from 15 protocols to determine the most reliable approach for estimating intake of HM leptin, adiponectin, insulin, glucose, and total lipid. Twenty mothers who were 3–5 months postpartum completed a 24 h milk profile study with pre-/post-feed milk samples collection. The true infant intake (control group) based on 24 h milk intake (MI) was compared to estimated infant intakes using concentrations from five sampling protocols that were multiplied by one of true infant MI, considered mean MI (800 mL), or global mean MI (766 mL). The mean measured concentrations of six samples (three sets of pre- and post-feed samples, from morning (06:00–09:00), afternoon (13:00–16:00), and evening (19:00–22:00)) multiplied by the true infant MI, mean considered MI, and global mean MI produced the most accurate estimates of infant intake of these components. Therefore, in the absence of 24 h measurements and sampling, a sampling protocol comprising three sets of pre-/post-feed samples provides the most reliable infant intake estimates of HM leptin, adiponectin, insulin, glucose, and total lipid.

## 1. Introduction

Human lactation research has been conceptualized as a biological framework in which human milk (HM), in terms of composition, is impacted by environmental factors, including maternal and infant factors [1,2,3,4,5,6]. During the last decade, HM has become a public health focus, considering the health benefits of breastfeeding for infant growth and health programming [7,8,9,10,11]. Human milk is a dynamic complex fluid in which many components, such as leptin, adiponectin, insulin, glucose, and total lipid concentrations differ within a feed and/or throughout the day [12,13,14].

Recent evidence indicates that HM leptin, adiponectin, insulin, glucose, and total lipid have circadian rhythms; thus, the timing of sample collection must be considered when exploring relationships of HM components with maternal factors or infant outcomes [12]. Currently, one of the most comprehensive methods of measuring infant intake of HM components is to use measured concentrations of all pre- and post-feed samples collected over a 24 h period while using electronic scales to calculate the infant’s intake for each feed [15]. Intakes are then summed to provide the total intake of each component over a 24 h period [16]. Knowledge of time effects on sampling for component concentrations is still lacking for many HM components. Sampling HM in the morning is a common procedure which may or may not directly follow maternal overnight fasting [17,18,19,20,21,22]. For these reasons, it is feasible that sampling at one time point may be insufficient when investigating the impact of HM components on infant outcomes. A study of HM lipid indicates that accurate estimation of infant HM lipid intake is challenging due to the variability in lipid concentrations and feed volumes [23,24]. Comparing different HM sampling protocols, George et al. [23] suggested that using six samples (three sets of pre-and post-feed samples) collected over 24 h provides the most accurate estimate of lipid intake when it is not possible to perform 24 h test weighing (24 h milk profile) and that the potential inaccuracies of sampling protocols should be taken into consideration when interpreting infant lipid intake estimations.

Thus, estimating infant intake of milk components requires an appropriate sampling protocol and an accurate measurement or estimation of milk volume ingested by infants. HM studies use a diverse range of sampling protocols, including but not limited to a morning sample, morning pre- and post-feed samples, and 24 h milk sample collection [25,26]; many studies do not account for the throughout-the-day or within-feed variation. In addition, the volume of milk delivered to the infant can vary; it can be most accurately measured either using deuterium dilution or a 24 h milk profile (test weighing) method [15,27]. However, the application of 24 h milk intake measurements is often challenging in clinical or research practices. Therefore, established reference ranges could be used to estimate the intake of HM components by the infants. Recently, a global average of infant HM intake was provided by a comprehensive systematic review and meta-analysis which focused on reportedly healthy mothers and infants and validated reference methods for measuring milk intake [28]. More importantly, a global mean 24 h milk intake of 766 mL based on healthy, term, exclusively breastfed infants less than 6 months of age was reported. Additionally, the 24 h milk intake recommendation from the European Food Safety Authority Panel for exclusively breastfed infants aged 1–6 months is 800 mL, based on mean intakes from several reference studies conducted in 12 countries [29].

In this study, for the first time, we compared true measured infant intakes of HM leptin, adiponectin, insulin, glucose, and total lipid calculated using the 24 h milk sampling profile data with 24 h intake estimates calculated using an extensive range of sampling protocols and a true infant 24 h milk intake, and two references of 24 h infant milk intake values, a considered mean 24 h milk intake of 800 mL [29], and a global mean 24 h milk intake of 766 mL [28].

## 2. Materials and Methods

### 2.1. Study Participants and Study Design

Twenty mothers of term-born singleton infants, non-smokers, with no pregnancy complications, who exclusively breastfed [30] their infants, participated in this study at 3–5 months postpartum between August and December 2019. Maternal and infant demographics were recorded, 24 h milk profiles were measured by mothers at home, and pre-/post-feed HM samples were collected. For a period of 24 h, mothers weighed their infant using accurate digital baby weigh scales (BabyWeigh™, Medela Inc., McHenry, IL, USA, resolution 2 g, accuracy ± 0.034%) before and after each feed [31]. Weights of HM feeds were converted to volumes using the HM density of 1.03 g/mL [32]. Mothers also collected 1–2 mL of milk before and after each breastfeed and placed the samples into 5 mL polypropylene vials (Disposable Products, Adelaide, SA, Australia). The collected samples were labelled and immediately stored in the participant’s home freezer, then transported on ice to the laboratory at The University of Western Australia and kept at −20 °C until analysis. All mothers provided informed consent to participate in the study, which was approved by the Human Research Ethics Committee, The University of Western Australia (RA/4/20/6498).

### 2.2. Biochemical Analysis of Human Milk Components

Milk samples were prepared for biochemical analysis (leptin, adiponectin, insulin, and glucose) by thawing the HM samples at room temperature and then homogenized using beads homogenizer (BeadBugTM 6 Position Homogenizer (Benchmark Scientific, Sayreville, NJ, USA) at 3 cycles of 5 s each. After homogenization, samples were aliquoted into 0.5 mL polypropylene microcentrifuge tubes (SSIbio, Lodi, CA, USA) with the required volumes to measure the HM components of interest in duplicate. Analyses were performed for all collected samples as previously described [12]. Leptin concentration in whole HM samples was analyzed using the Human Leptin ELISA DuoSet (DY398, Lot: P262874, R & D system, Minneapolis, MN, USA). Adiponectin concentration in whole HM samples was analyzed using the Human Adiponectin ELISA, High Sensitivity (RD191023100, Lot: E21-040, BioVendor, Brno, Czech Republic). Insulin concentration in whole HM samples was analyzed using the human Insulin ELISA BioVendor (RIS006R, Lot: X21-136S01, BioVendor, Brno, Czech Republic). Glucose concentration in skim HM samples [12] was analyzed using a D-Glucose HK Assay Kit (K-GLUHK-220A, Lot: 200218-8, Megazyme, Wicklow, Ireland). Total lipid concentration (%) was measured using the creamatocrit method [33,34] and converted to g/L using the following formula:

Fat (g/L) = 3.968 + (5.917 × Creamatocrit (%))(1)

### 2.3. Calculation of Infant Intake of Human Milk Components 

The infants’ true intakes of HM leptin, adiponectin, insulin, glucose, and total lipid were calculated by averaging the measured pre-and post-feed concentrations and multiplying by the HM intake for the corresponding feed, then summing the component intakes for all feeds in the 24 h period (Protocol 1A, Table 1). Infant intake estimates were calculated using 15 different intake estimation protocols (Protocols 2B–4F, Table 1), designed to be representative of five sampling protocols used in previous studies. The following milk intake values were used for estimation of HM components intakes: the true 24 h milk intake of each infant; the European Food Safety Authority (EFSA) considered mean milk intake of 800 mL (taken as the basis for calculation of required nutrient intakes via HM and based on several mean intakes of healthy, term, exclusively breastfed infants less than 6 months of age measured in 12 countries) [29], and a global mean milk intake of healthy exclusively breastfed infants of 766 mL (3–5.9 months postpartum, *n* = 2619) [28] measured with validated reference methods of test weighing and deuterium dilution [27]. 

### 2.4. Statistical Analysis

Statistical analysis was performed using R statistical software, version 4.3.1 [35]. The packages lme4 [36], lmerTest [37], and emmeans [38] were used for linear mixed-model analysis, and the packages ggplot2 [39] and ggpubr [40] were used for data visualization. Data are presented as mean ± standard deviation (SD) unless specified. The 24 h HM component intake estimate for each sampling protocol was compared against the measured 24 h HM component intake that was considered as a control group (true intake). Linear mixed models were used to compare the mean intake resulting from each sampling protocol for response variables, with protocol included as a fixed effect and the mother as a random effect. Standard graphical methods were used to check the models’ assumptions and variables were transformed if needed to meet the assumptions of the statistical test. Results are presented as mean ± SD, parameter estimate and 95% CIs for comparisons. *p*-values < 0.05 were considered significant.

## 3. Results

### 3.1. Participant Characteristics

Twenty breastfeeding mothers of infants born at normal weight provided 24 h milk profile data and collected samples pre- and post-feed. Of the twenty mothers, eleven measured their infant’s milk intake at 3 months, three at 4 months, and six at 5 months postpartum. The participants’ demographics and breastfeeding characteristics are presented in Table 2.

### 3.2. Concentrations of Milk Components

Based on complete 24 h milk profile data and pre- and post-feed HM sample analyses, the mean 24 h concentrations of HM leptin, adiponectin, insulin, glucose, and total lipid are provided in Table 3. The mean concentrations of leptin, adiponectin, insulin, glucose, and total lipid for the 24 h samples were compared to those of the five sampling protocols (Table 4). No significant differences were detected for the adiponectin and leptin concentrations. Morning sampling protocols (1/pre/am, 1/post/am, and 2/1pre/1post/am) significantly underestimated the insulin concentrations. The sampling protocol using one post-feed sample collected in the morning (1/post/am) significantly underestimated the glucose concentration. The pre-feed sampling protocols (1/pre/am and 3/pre/24 h) underestimated whilst the post-feed sampling protocols (1/post/am) overestimated the total lipid concentrations in comparison to the mean concentration based on the 24 h sampling protocol (Table 4).

### 3.3. 24 h Measured Intake of Milk Components

**Figure 1:**
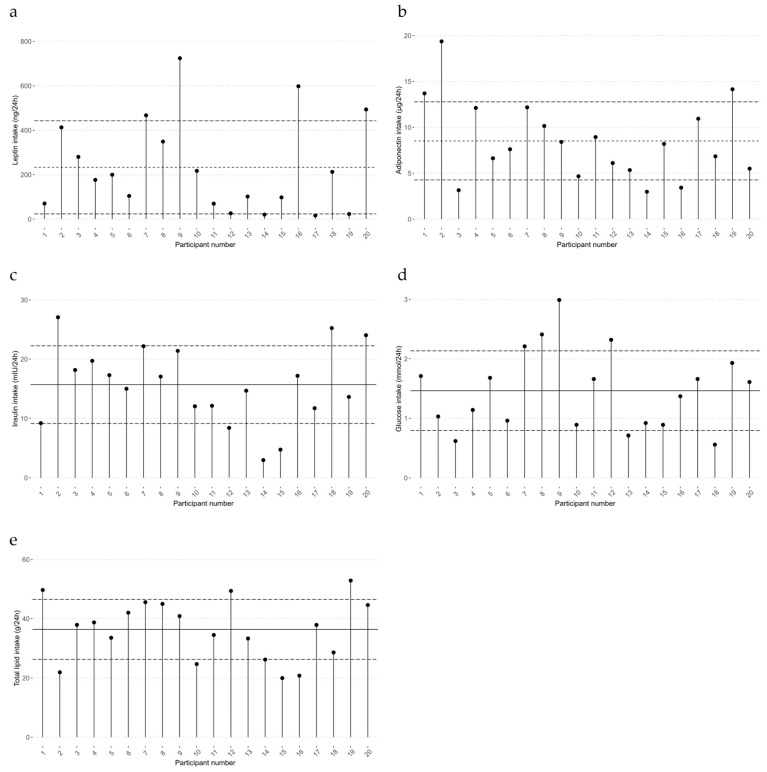
True infant 24 h intakes of human milk leptin (**a**), adiponectin (**b**), insulin (**c**), glucose (**d**), and total lipid (**e**). The intake of each component was calculated using 24 h milk profile data and samples. Mean (–) and standard deviation (--) are indicated.

The true infant intakes of HM components (protocol 1A) were as follows: leptin, 233.0 ± 2.9.6 ng/24 h; adiponectin, 8.5 ± 4.3 µg/24 h; insulin, 15.7 ± 6.6 µg/24 h; glucose, 1.5 ± 0.7 mmol/24 h; total lipid, 36.4 ± 10.1 g/24 h (Figure 1).

### 3.4. Estimated Intake of Milk Components

**Figure 2:**
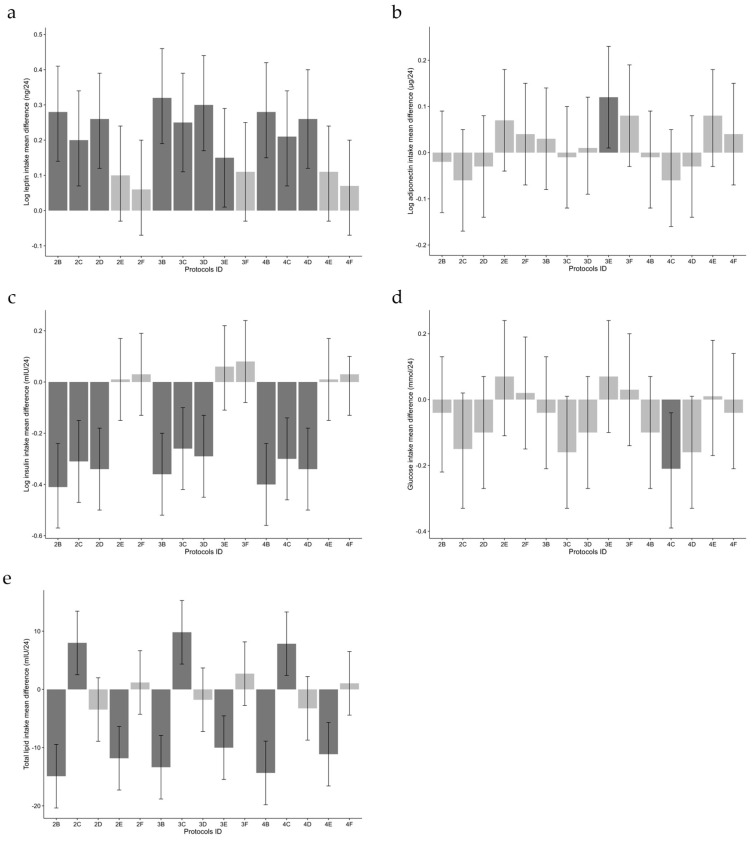
Mean difference with 95% confidence intervals of infant estimated intakes (2B–4F; see Table 1) compared to true intake (protocol 1A) of leptin (**a**), adiponectin (**b**), insulin (**c**), glucose (**d**), and total lipid (**e**). Dark grey indicates significant differences (*p* < 0.05) and light grey indicates no differences when compared to protocol 1A.

The true infant intakes of leptin, adiponectin, insulin, glucose, and total lipid were compared to the estimated intakes (Table 5). The intakes of each protocol and their coefficients are shown in Figure 2. Protocols 2B, 2C, 2D, 3B, 3C, 3D, 4B, 4C, and 4D significantly overestimated infant intake of leptin and underestimated the intake of insulin (Figure 2a,b). Protocols 2B, 2E, 3B, 3E 4B, and 4E significantly underestimated infant intake of total lipid, whilst protocols 2C, 3C, and 4C significantly overestimated the intake of total lipid (Figure 2e). Protocol 4C significantly underestimated infant intake of glucose (Figure 2d). Protocol 3E significantly overestimated infant intake of adiponectin (Figure 2b). Overall, the sampling protocol 6/3pre/3post/24 h was found to be the most accurate in estimating infant intakes of HM components when assumed (800 and 766 mL/24 h) or true measured 24 h HM intake volumes were used (protocols 4D, 4E and 4F) (Table 5 and Figure 2).

## 4. Discussion

This study found significant differences between the true and estimated intakes of HM components. The largest discrepancies were apparent for insulin, leptin, and total lipid when using the most common sampling method of a single morning sample. The discrepancies between the true and estimated HM component intakes were largely due to the infrequent concentration measures. For leptin and insulin, analysis of three to six HM samples collected across the 24 h period produced more accurate intake estimates, whether using test weighing to measure feed volumes or using assumed intakes, i.e., 800 mL/24 h or 766 mL/24 h.

The abovementioned findings of discrepancies in estimated HM component intakes are likely a reflection of circadian patterns of the component concentrations as with leptin and insulin [12] as well as concentration variability due to the fullness of the breast (e.g., total lipid) [24,41] and wide variations in 24 h milk intakes between infants [31]. The importance of accurate intake estimation has been anticipated in recent systematic reviews that highlighted that studies which investigate relationships between HM components and infant outcomes mainly measure concentrations often producing inconclusive results [42,43,44]. Further, studies of HM leptin and adiponectin showed that it was not concentrations, but higher 24 h intake of whole milk leptin that was related to increased infant adiposity [45], and higher adiponectin intake was associated with increased infant adiposity and anthropometrics [45,46] and reduced infant fat-free mass during the first year of life [45]. Only a few studies have investigated the link between the intake of HM components and infant growth outcomes and these show promise in elucidating the mechanisms by which HM components influence infant growth and development of body composition [42,47]. Therefore, the results of this study provide recommendations for sampling and intake estimation protocols that could be applied to investigate the relationships between HM components and infant outcomes in future studies.

Previous studies have emphasised the variability in HM total lipid intake (mainly due to its concentration variability throughout 24 h [12]), and the importance of more frequent sampling for accurate assessment of infant lipid intake over a 24 h period to understand its potential impact on infant growth and development [23,48,49]. George et al. demonstrated that an exclusively breastfed infant who was born small-for-gestational age had slow weight gain, despite the mother having a total HM lipid concentration within the reference range [48]. This reduced weight gain was attributed to the low 24 h milk intake, such that the infant’s calculated 24 h total lipid intake was below the reference range, thus contributing to low energy intake which resulted in poor infant growth. Thus, timely and accurate assessment of infant intake of lipid is crucial in determining adequacy of nutrition in cases of infants failing to thrive.

Infant milk intake has been long known to vary throughout 24 h as shown by test weighing [16]. Test weighing and deuterium dilution are two validated and highly correlated methods for determining infant milk intake [15,27]. Both methods are not without limitations, as both rely on participant compliance and can be costly. Test weighing requires high-accuracy electronic scales [27], whilst the deuterium dilution method does not provide information on the highly variable individual feed volumes [31,50]. A recent systematic review has approximated the mean HM intake volume for the exclusively breastfeeding infant aged 3–6 months to be 766 mL/24 h [28]. Variation between studies that measure milk volume may result from maternal compliance with measurement protocols, and errors in recording of measurements as well as the absence of documentation of feeds other than HM. In our cohort, when comparing the true component intake with estimated intakes from different sampling protocols, we found that using pre- or post-feed morning samples resulted in inaccurate intake estimations of HM leptin, insulin, and total lipid. These differences remained consistent whether using actual 24 h volume intake (true intake for each infant), 800 mL/24 h, or 766 mL/24 h for estimating infant intakes of leptin, insulin, and total lipid. Therefore, we consider that the sampling method is a key element for accurate estimation of infant HM component intake; when a 24 h sampling profile is not possible, the most reliable alternative sampling protocol is the use of six samples (three sets of pre- and post-feed samples) throughout the 24 h (6/3pre/3post/24 h).

Our data highlighted that some components, such as adiponectin and glucose, might not need multiple sample collection throughout 24 h to accurately estimate their intake by the infant, thus HM sampling protocols could be tailored to the research question and study type. However, currently there are rationale and a critical need to uncover the different functions of the components in HM within the concept of HM as a biological system. Therefore, a proper sampling that could be utilized to measure a number of HM components per sample could provide an opportunity to study HM in its complexity, and this may be considered as a reductionist approach.

While we provide a future direction for the design of HM studies in terms of sampling and estimating infant HM component intakes, these results cannot be generalized to be used in some populations, due to the substantial differences in milk intake between infants (Figure 1), particularly in those of women with low milk supply. The results from the case study by George et al. [48] highlight that there is a possibility of overestimation of intake of HM components such as total lipid, protein, and lactose concerning for infant health outcomes. Therefore, the estimation protocols presented in this study might not be an appropriate replacement for the objective HM components intake measurement.

One potential limitation is that while relevant to the established lactation period when milk intake is consistent [51], this intake estimation method cannot be applied to the first week postpartum during the establishment of full milk production due to delays in some mothers reaching their full capacity, or after the introduction of solid food, when milk volume intakes start to diminish. Studies of HM intake showed an increase from 29 mL during the first 24 h postpartum to approximately 415 mL by day 5, reaching 680 mL by day 8 postpartum [52]. Further, as concentrations of some HM components, such as leptin, adiponectin, and insulin are known to decrease over the exclusively breastfeeding period [53,54,55,56], then predictions of their intakes may not be accurate. This should be accounted for when trying to decipher the impact on infant outcomes. Indeed, Rios-Leyvraz and Yao [28] suggest that milk intake over time can be modelled as follows: milk intake (mL/24 h) = 51−1.4 × days +180 × log(days). However, this formula has yet to be validated with measured data, and it conflicts with evidence that during established lactation (1–6 months) infants generally have no change in 24 h milk intake once a full milk production is established [51].

This study represents an original contribution to the field of HM research, provides direction for further research in terms of sampling protocols for measurement of HM component intakes, and demonstrates accurate alternative sampling protocols to improve the experimental framework and analytical approaches to HM component studies. In addition, using the estimation protocol (6/3pre/3post/24 h) suggested in our study might facilitate the investigation of longitudinal variations in infant intake of HM components and their impact on infant growth and development in larger longitudinal human milk studies.

## 5. Conclusions

In view of the current approach to HM as a biological system, a systematic comparison of different protocols to estimate infant intake of HM components showed that collecting multiple milk samples, pre- and post-feed, and across the day (morning, day, and evening) might be an important consideration when designing a study to investigate the effect of HM component intakes on infant outcomes; in particular, for those components that exhibit a circadian or within-feed variation, a sampling protocol that is closest to the true measure of component intake should be considered to provide the most accurate estimate. 

## Figures and Tables

**Table 1:** Description of sampling and component intake measurements protocols.

Protocol ID	Sampling	Milk Intake Used in Calculation
1A	True intake: all pre- and post-feed samples throughout 24 h	Milk intake for each feed throughout 24 h, mL
2B	1/pre/am/true: pre-feed morning sample (06:00–10:00)	True milk intake per infant, mL/24 h
2C	1/post/am/true: post-feed morning sample (06:00–10:00)	
2D	2/1pre/1post/am/true: mean of pre- and post-feed morning samples (06:00–10:00)	
2E	3/pre/24 h/true: mean of 3 pre-feed samples, morning (06:00–09:00), afternoon (13:00–16:00), evening (19:00–22:00)	
2F	6/3pre/3post/24 h/true: mean of 6 samples, pre- and post-feed, morning (06:00–09:00), afternoon (13:00–16:00), evening (19:00–22:00)	
3B	1/pre/am/800: pre-feed morning sample (06:00–10:00)	800 mL/24 h ^a^
3C	1/post/am/800: post-feed morning sample (06:00–10:00)	
3D	2/1pre/1post/am/800: mean of pre- and post-feed morning samples (06:00–10:00)	
3E	3/pre/24 h/800: mean of 3 pre-feed samples, morning (06:00–09:00), afternoon (13:00–16:00), evening (19:00–22:00)	
3F	6/3pre/3post/24 h/800: mean of 6 samples, pre- and post-feed, morning (06:00–09:00), afternoon (13:00–16:00), evening (19:00–22:00)	
4B	1/pre/am/766: pre-feed morning sample (06:00–10:00)	766 mL/24 h ^b^
4C	1/post/am/766: post-feed morning sample (06:00–10:00)	
4D	2/1pre/1post/am/766: mean of pre- and post-feed morning samples (06:00–10:00)	
4E	3/pre/24 h/766: mean of 3 pre-feed samples, morning (06:00–09:00), afternoon (13:00–16:00), evening (19:00–22:00)	
4F	6/3pre/3post/24 h/766: mean of 6 samples, pre-feed and post-feed, morning (06:00–09:00), afternoon (13:00–16:00), evening (19:00–22:00)	

^a^ European Food Safety Authority (EFSA) mean of human milk volume for calculation of infant nutrient intakes [29]. ^b^ Global mean of human milk intake of exclusively breastfed infants between 3 and 6 months postpartum [28]. Abbreviations: h, hour; am, morning; post, post-feed; pre, pre-feed.

**Table 2:** Participant characteristics.

Characteristics	*n* = 20
Maternal	
Age (years)	32.7 ± 5.3 (25–46) ^a^
BMI (kg/m^2^)	27.3 ± 6.3 (19.2–38.7)
Infant	
Sex (male, female)	12, 8
Gestational age (weeks)	38.9 ± 1.4 (36–41)
Birth weight (g)	3447 ± 382 (2940–4455)
Breastfeeding characteristics	
Time postpartum (months)	3.8 ± 0.9 (3.0–5.1)
24 h milk intake (mL)	791 ± 212 (441–1180)
Feeding frequency ^b^	12.7 ± 2.6 (7–16)

^a^ Mean ± SD and (Min–Max). ^b^ Feeding defined as breastfeed from one breast.

**Table 3:** Concentrations of human milk components.

	*n*	Mean ± SD	Median	Q1	Q3	IQR	Min–Max
Leptin (pg/mL)	427	308.69 ± 270.30	238.27	87.08	478.91	391.83	14.9–1285.0
Adiponectin (ng/mL)	453	10.95 ± 5.11	9.90	7.13	13.55	6.42	3.4–33.1
Insulin (µIU/mL)	436	20.59 ± 11.27	19.39	11.98	27.68	15.69	1.3–76.1
Glucose (mmol/L)	445	1.85 ± 0.73	1.84	1.25	2.36	1.11	0.28–4.0
Total lipid (g/L)	439	47.49 ± 23.00	44.20	28.23	63.14	34.91	9.9–134.1

The milk component concentrations (mean ± standard deviation) based on all feeds throughout the 24 h period. Abbreviations: IQR, interquartile range; Min, minimum, Max, maximum.; Q1, quartile 1; Q3, quartile 3.

**Table 4:** Difference in mean human milk component concentrations using various sampling protocols.

Sampling Protocol	Estimates	95% CI	*p* ^b^
Log leptin, pg/mL			
24 h average ^a^	5.34	4.85, 5.82	-
1/pre/am	0.14	0.14, 0.41	0.08
1/post/am	0.07	0.07, 0.34	0.4
2/1pre/1post/am	0.12	0.12, 0.39	0.14
3/pre/24 h	−0.03	−0.03, 0.24	0.68
6/3pre/3post/24 h	−0.07	−0.07, 0.20	0.37
Log adiponectin, ng/mL			
24 h average ^a^	2.30	2.09, 2.51	-
1/pre/am	−0.03	−0.10, 0.05	0.45
1/post/am	−0.07	−0.15, 0.00	0.06
2/1pre/1post/am	−0.04	−0.12, 0.03	0.24
3/pre/24 h	0.06	−0.01, 0.14	0.10
6/3pre/3post/24 h	0.03	−0.05, 0.10	0.50
Log insulin, µIU/mL			
24 h average ^a^	3.06	2.78, 3.35	-
1/pre/am	−0.56	−0.75, −0.37	**<0.001**
1/post/am	−0.46	−0.65, −0.27	**<0.001**
2/1pre/1post/am	−0.49	−0.69, −0.30	**<0.001**
3/pre/24 h	−0.15	−0.34, 0.05	0.13
6/3pre/3post/24 h	−0.12	−0.31, 0.07	0.20
Glucose, mmol/L			
24 h average ^a^	0.55	0.37, 0.72	-
1/pre/am	−0.06	−0.16, 0.04	0.24
1/post/am	−0.11	−0.21, −0.01	**0.03**
2/1pre/1post/am	−0.07	−0.17, 0.03	0.16
3/pre/24 h	0.03	−0.07, 0.14	0.49
6/3pre/3post/24 h	0.01	−0.09, 0.12	0.77
Total lipid, g/L			
24 h average ^a^	48.02	41.93, 54.11	-
1/pre/am	−19.2	−25.09, −13.31	**<0.001**
1/post/am	9.76	3.87, 15.65	**0.001**
2/1pre/1post/am	−4.72	−10.61, 1.17	0.11
3/pre/24 h	−15.0	−20.89, −9.11	**<0.001**
6/3pre/3post/24 h	0.88	−5.01, 6.77	0.76

Data for different sampling protocols compared to the 24 h sampling profile samples. Differences are shown as parameter estimates and 95% CI. Abbreviation: am, morning; CI, coefficient interval; 24 h, mean of samples taken from morning, afternoon, and evening; post, post-feed sample; pre, pre-feed sample. ^a^ The 24 h human milk concentrations calculated as the mean of all pre- and post-feed sample concentrations for a 24 h period. ^b^ *p* value indicates a significant difference (*p* < 0.05) compared with true mean human milk component concentration (**indicated in bold font**).

**Table 5:** Intakes of human milk components using true and estimated milk intakes volumes.

	True Milk Intake ^c^				800 mL/24 h ^d^				766 mL/24 h ^e^			
Sampling Protocol	Mean ± SD	Estimates	95% CI	*p* ^f^	Mean ± SD	Estimates	95% CI	*p* ^f^	Mean ± SD	Estimates	95% CI	*p* ^f^
Log leptin, ng/24 h ^a^												
True intake ^b^	4.93 ± 1.18	-	4.44, 4.52	-	-	-	-	-	-	-	-	-
1/pre/am	5.21 ± 1.11	0.28	0.14, 0.41	**<0.001**	5.25 ± 1.10	0.32	0.19, 0.46	**<0.001**	5.21 ± 1.11	0.28	0.15, 0.42	**<0.001**
1/post/am	5.13 ± 1.04	0.20	0.07, 0.34	**0.004**	5.18 ± 1.07	0.25	0.11, 0.39	**<0.001**	5.14 ± 1.07	0.21	0.07, 0.37	**0.003**
2/1pre/1post/am	5.19 ± 1.04	0.26	0.12, 0.39	**<0.001**	5.23 ± 1.06	0.30	0.17, 0.44	**<0.001**	5.19 ± 1.06	0.26	0.12, 0.40	**<0.001**
3/pre/24 h	5.03 ± 1.14	0.10	−0.03, 0.24	0.13	5.08 ± 1.13	0.15	0.01, 0.29	**0.03**	5.04 ± 1.14	0.11	−0.03, 0.24	0.12
6/3pre/3post/24 h	4.99 ± 1.14	0.06	−0.07, 0.20	0.36	5.04 ± 1.14	0.11	−0.03, 0.25	0.11	5.00 ± 1.14	0.07	−0.07, 0.20	0.33
Log adiponectin, µg/24 h ^a^												
True intake ^b^	2.02 ± 0.52	-	1.79, 2.25	-	-	-	-	-	-	-	-	-
1/pre/am	2.00 ± 0.64	−0.02	−0.13, 0.09	0.75	2.05 ± 0.51	0.03	−0.08, 0.14	0.59	2.01 ± 0.51	−0.01	−0.12, 0.09	0.80
1/post/am	1.96 ± 0.63	−0.06	−0.17, 0.05	0.27	2.01 ± 0.49	−0.01	−0.12, 0.10	0.81	1.96 ± 0.49	−0.06	−0.16, 0.05	0.30
2/1pre/1post/am	1.99 ± 0.63	−0.03	−0.14, 0.08	0.55	2.03 ± 0.49	0.01	−0.09–0.12	0.79	1.99 ± 0.49	−0.03	−0.14, 0.08	0.59
3/pre/24 h	2.09 ± 0.57	0.07	−0.04, 0.18	0.18	2.14 ± 0.44	0.12	0.01–0.23	**0.03**	2.10 ± 0.44	0.08	−0.03, 0.18	0.16
6/3pre/3post/24 h	2.06 ± 0.57	0.04	−0.07, 0.15	0.50	2.10 ± 0.44	0.08	−0.03, 0.19	0.13	2.06 ± 0.44	0.04	−0.07, 0.15	0.46
Log insulin, mIU/24 h ^a^												
True intake ^b^	2.64 ± 0.55	-	2.35, 2.92	-	-	-	-	-	-	-	-	-
1/pre/am	2.23 ± 0.81	−0.41	−0.57, −0.24	**<0.001**	2.28 ± 0.80	−0.36	−0.52, −0.20	**<0.001**	2.23 ± 0.80	−0.40	−0.56, −0.24	**<0.001**
1/post/am	2.33 ± 0.72	−0.31	−0.47, −0.15	**<0.001**	2.38 ± 0.67	−0.26	−0.42, −0.10	**0.002**	2.33 ± 0.67	−0.30	−0.46, −0.14	**<0.001**
2/1pre/1post/am	2.30 ± 0.73	−0.34	−0.50, −0.18	**<0.001**	2.34 ± 0.70	−0.29	−0.45, −0.13	**<0.001**	2.30 ± 0.70	−0.34	−0.50, −0.18	**<0.001**
3/pre/24 h	2.65 ± 0.52	0.01	−0.15, 0.17	0.91	2.69 ± 0.49	0.06	−0.11, 0.22	0.50	2.65 ± 0.49	0.01	−0.15, 0.17	0.66
6/3pre/3post/24 h	2.67 ± 0.51	0.03	−0.13, 0.19	0.70	2.72 ± 0.46	0.08	−0.08, 0.24	0.34	2.67 ± 0.46	0.03	−0.13, 0.20	0.87
Glucose, mmol/24 h ^a^												
True intake ^b^	1.46 ± 0.67	-	1.22, 1.71	-	-	-	-	-	-	-	-	-
1/pre/am	1.42 ± 0.71	−0.04	−0.22, 0.13	0.61	1.42 ± 0.52	−0.04	−0.21, 0.13	0.65	1.36 ± 0.50	−0.10	−0.27, 0.07	0.25
1/post/am	1.31 ± 0.57	−0.15	−0.33, 0.02	0.07	1.30 ± 0.38	−0.16	−0.33, 0.01	0.07	1.25 ± 0.37	−0.21	−0.39, −0.04	**0.01**
2/1pre/1post/am	1.36 ± 0.63	−0.10	−0.27, 0.07	0.25	1.36 ± 0.44	−0.10	−0.27, 0.07	0.25	1.31 ± 0.42	−0.16	−0.33, 0.01	0.07
3/pre/24 h	1.53 ± 0.57	0.07	−0.11, 0.24	0.44	1.53 ± 0.56	0.07	−0.10, 0.24	0.41	1.47 ± 0.53	0.01	−0.17, 0.18	0.94
6/3pre/3post/24 h	1.48 ± 0.67	0.02	−0.15, 0.19	0.83	1.49 ± 0.51	0.03	−0.14, 0.20	0.75	1.43 ± 0.49	−0.04	−0.21, 0.14	0.67
Total Lipid, g/24 h ^a^												
True intake ^b^	36.4 ± 10.1	-	31.5, 41.3	-	-	-	-	-	-	-	-	-
1/pre/am	21.5 ± 7.35	−14.9	−20.4, −9.44	**<0.001**	23.1 ± 9.06	−13.4	−18.8, −7.90	**<0.001**	22.1 ± 8.68	−14.34	−19.8, −8.88	**<0.001**
1/post/am	44.4 ± 17.6	7.99	2.53, 13.4	**0.004**	46.2 ± 17.8	9.81	4.35, 15.27	**<0.001**	44.3 ± 17.0	7.84	2.39, 13.3	**0.005**
2/1pre/1post/am	33.0 ± 10.8	−3.46	−8.91, 2.00	0.21	34.6 ± 11.9	−1.78	−7.23, 3.68	0.52	33.2 ± 11.4	−3.25	−8.71, 2.21	0.24
3/pre/24 h	24.6 ± 5.58	−11.8	−17.3, −6.36	**<0.001**	26.4 ± 8.06	−10.0	−15.5, −4.54	**<0.001**	25.3 ± 7.72	−11.1	−16.6, −5.67	**<0.001**
6/3pre/3post/24 h	37.6 ± 10.3	1.19	−4.27, 6.65	0.66	39.1 ± 8.77	2.70	−2.76, 8.16	0.33	37.5 ± 8.40	1.04	−4.42, 6.50	0.70

Data for estimation protocols (B–P) compared to the true intake protocol (A) (see Table 1). Differences are shown as parameter estimates and 95% CI. Abbreviations: am, morning; CI, coefficient interval; 24 h, mean of samples taken from morning, afternoon, and evening; post, post-feed sample; pre, pre-feed sample. ^a^ The mean infant intake of each protocol presented as mean ± SD. ^b^ True human milk intake calculated as the sum of all pre- and post-feed samples concentrations of a 24 h period multiplied by the corresponding feed volume. ^c^ The measured true milk intake of each infant (mL/24 h) used to calculate infants’ intake for each sampling protocol. ^d^ The EFSA mean considered milk intake of 800 mL/24 h used to calculate infants’ intake for each sampling protocol [29]. ^e^ The global mean milk intake of exclusively breastfed infants of 766 mL/24 h used to calculate infants’ intake for each sampling protocol [28]. ^f^ *p* value indicates a significant difference (*p* < 0.05) compared with true HM component intake (**indicated in bold font**).

## Data Availability

The data presented in this study are available from the corresponding author upon reasonable request.
